# Periodontitis Induced by *P. gingivalis*-LPS Is Associated With Neuroinflammation and Learning and Memory Impairment in Sprague-Dawley Rats

**DOI:** 10.3389/fnins.2020.00658

**Published:** 2020-07-02

**Authors:** Yi Hu, Huxiao Li, Jing Zhang, Xu Zhang, Xinyi Xia, Che Qiu, Yue Liao, Huiwen Chen, Zhongchen Song, Wei Zhou

**Affiliations:** ^1^Department of Periodontology, Shanghai Ninth People’s Hospital, College of Stomatology, Shanghai Jiao Tong University School of Medicine, Shanghai, China; ^2^National Clinical Research Center for Oral Diseases, Shanghai Key Laboratory of Stomatology & Shanghai Research Institute of Stomatology, Shanghai, China; ^3^Laboratory of Oral Microbiota and Systemic Diseases, Shanghai Ninth People’s Hospital, College of Stomatology, Shanghai Jiao Tong University School of Medicine, Shanghai, China

**Keywords:** periodontitis, neuroinflammation, learning and memory impairment, APP processing, TLR4/NF-κB signaling pathway

## Abstract

**Background:**

Periodontitis is one of the most common oral diseases and is a potential risk factor for systemic diseases. In this study, we aimed to investigate the association between periodontitis and learning and memory impairment.

**Methods:**

We established a periodontitis model by topical application of *Porphyromonas gingivalis* lipopolysaccharide (*P. gingivalis*-LPS) into the palatal gingival sulcus of the maxillary first molars of 10-week-old male rats for a 10-week period. We assessed alveolar bone resorption using micro–computed tomography analysis and learning and memory ability using the Morris water maze test. We determined the levels of cytokines [interleukin (IL)-1β, IL-6, IL-8, and IL-21] and LPS in the peripheral blood and cortex, as well as toll-like receptor 4 (TLR4)/NF-κB signaling pathway activation, using reverse transcription-polymerase chain reaction (RT-PCR), enzyme-linked immunosorbent assay (ELISA), and western blot. We determined activation of microglia and astrocytes, expression of Aβ1-42, APP and Tau by immunohistochemistry. Finally, we measured the expression of amyloid precursor protein (APP) and its key secretases, as well as the Aβ1-40/1-42 ratio, by RT-PCR, western blot, and ELISA.

**Results:**

We found that periodontitis induced learning and memory impairment in the rats. Further, we observed that it induced significant alveolar bone resorption. There was an increase in the levels of inflammatory cytokines and LPS. Moreover, we confirmed TLR4/NF-κB signaling pathway activation. We also observed activated microglia and astrocytes with enlarged cell bodies and irregular protrusions. Finally, we observed the promotion of β- and γ-secretases APP processing.

**Conclusion:**

Our findings indicated that periodontitis was associated with learning and memory impairment, probably induced by neuroinflammation via activating the TLR4/NF-κB signaling pathway. Furthermore, abnormal APP processing could be involved in this progress.

## Introduction

Periodontitis is a potentially transmissible chronic infection caused by plaque biofilm. It is responsible for 70% of the global population presenting one or more damages in the periodontium (Oppermann et al., 2015). Considering that periodontitis involves interacting bacterial pathogens, including their toxic factors and host inflammatory responses, it has a multifactorial etiology with a marked inflammatory profile (Papapanou et al., 2018). Chronic recurrent periodontal inflammation could destroy the tooth support organization; further, there is increasing evidence that periodontal disease is an important risk factor for systemic diseases, including cardiovascular disease, diabetes, and reduced respiratory function (Preshaw et al., 2012; Hamilton et al., 2017; Winning et al., 2019).

Given the accelerating trend of population aging, the harm caused by cognitive disorders on human health has become increasingly prominent. It clinically manifests as gradual cognitive dysfunction and psychosis (Wimo et al., 2017; Brookmeyer et al., 2018). Epidemiological surveys reported that periodontitis was associated with an increase in the rate of cognitive decline (Ide et al., 2016; Harding et al., 2017). Periodontitis is not only an oral localized inflammatory disease, but also elicits low-grade inflammation via both the release of pro-inflammatory cytokines and the invasion of periodontitis bacteria along with their components (Hashioka et al., 2019). Level of pro-inflammatory cytokines could increase with worsened periodontal status and cognitive decline (Sochocka et al., 2017). Keeping oral hygiene could decrease the risk of dementia (Harding et al., 2017).

Periodontal pathogens and virulence factors that cause chronic periodontitis may also possibly contribute to the development of dementia (Olsen and Singhrao, 2015). *Porphyromonas gingivalis (P. gingivalis)*, the most important periodontal pathogen, and its virulence factor lipopolysaccharide (LPS) are main pathogenic factor of periodontitis (Jain and Darveau, 2010; Poole et al., 2013). Dominy et al. (2019) reported *P. gingivalis* in brain samples of patients with AD, which indicates that it could be a risk factor for AD. Ilievski et al. (2018) reported brain inflammation and neurodegeneration after oral administration of *P. gingivalis*. Wu et al. (2017) reported that intraperitoneal injection of *P. gingivalis*-LPS promoted learning and memory deficits and intracellular accumulation of Aβ. Our previous study also suggested that intraperitoneal injection of *P. gingivalis*-LPS, which induced systemic inflammation, could impair cognition through neuroinflammation (Zhang et al., 2018). However, the association between topical application of *P. gingivalis*-LPS into the gingival sulcus and learning and memory ability remains unclear.

In this study, we aimed to investigate the effects of periodontitis with topical application of *P. gingivalis*-LPS on learning and memory using the Morris Water Maze test. We used micro–computed tomography to assess bone resorption. Activation of microglial and astrocyte was confirmed through immunohistochemistry. The relative level of LPS and expression of inflammatory factors, as well as TLR4/NF-κB signaling pathway activation were analyzed using reverse transcription-polymerase chain reaction, enzyme-linked immunosorbent assay, and western blot. For further analysis of the underlying mechanism, we assessed APP processing.

## Materials and Methods

### Animals

All experimental protocols were approved by the ethical committee of the Animal Care and Experimental Committee of Shanghai Jiao Tong University School of Medicine and were performed according to the guidelines of the EU Directive 2010/63/EU. Efforts were made to minimize surgery-induced suffering and reduce the overall number of animals used.

We obtained 10-week-old male Sprague-Dawley rats of specific pathogen-free (SPF) grade from the Shanghai SIPPR-BK Laboratory Animal Co., Ltd. The rats were provided with standard housing at temperature (18–22°C) and humidity (55–65%) with a 12-h light/dark cycle and free access to food and water. We randomly divided 32 rats into four groups, namely, the control group (Control), *P. gingivalis*-LPS group (LPS), *P. gingivalis*-LPS plus TAK-242 group (LPS+TAK-242), and TAK-242 group. TAK-242 (also known as CLI-095), a novel cyclohexene derivative, is an effectively/specifically TLR4 inhibitor (Ii et al., 2006). After inducing anesthesia using 10% chloral hydrate, the rats in the LPS and LPS+TAK-242 groups were topical applied with *P. gingivalis*-LPS (0.5 mg/kg, twice a week) into the palatal gingival sulcus of the maxillary first molars for 10 weeks using a 5-μl microsyringe (Hamilton, Switzerland). Moreover, we injected TAK-242 (0.5 mg/kg, i.p., twice a week) in rats in the LPS+TAK-242 group in advance. The rats in the TAK-242 group were injected with TAK-242 (0.5 mg/kg, i.p., twice a week). The rats in the control group received an equivalent saline volume. The LPS and TAK-242 amounts were determined based on previous studies (Nishida et al., 2001; Gárate et al., 2014). Three days after the last administration, the rats underwent behavioral tests for learning and memory ability assessment. We purchased *P. gingivalis*-LPS Ultrapure and TAK-242 from Invivogen (tlrl-ppglps and tlrl-cli95, San Diego, CA, United States) and dissolved them according to the manufacturer’s instructions.

### Open Filed Test (OFT)

The open field in the present study consisted of a rectangular arena (530 mm × 478 mm), enclosed by a black wall, 590 mm in height (Mobile Datum, Shanghai, China). The test was initiated by gently placing a single rat in the middle of the arena, allowing the animal to move freely for 5 min while being recorded.

### Morris Water Maze (MWM) Test

The MWM test was conducted in a round pool 160 cm in diameter and 55 cm in depth (Mobile Datum, Shanghai, China). The pool was filled with water made opaque with white non-toxic water-based tempura paint. The water temperature was controlled to remain with a range equivalent to that of room temperature (22 ± 1°C). The platform was placed in the center of one quadrant of the pool and submerged 2.5 cm beneath the water surface; it remained in the same position throughout the learning trials and was removed from the pool during the probe test. A video-tracking system (Shanghai Jiliang Software Technology Co., Ltd.) was used to monitor and record the swimming activity of the rats. The rats should have learned to use the visual tips around the pool to find the hidden platform within 90 s, otherwise it would be gently guided to the platform and allowed to re-orient for an additional 10 s. Each rat was trained four times per day with a 30 s of rest per training interval. To examine spatial reference memory, a probe test was carried out on the sixth day when the platform was removed from the pool and each rat was placed into the water at the two quadrants furthest from the platform used on days 1–5, being allowed to navigate freely for 60 s.

### Measurement of Alveolar Bone Resorption by Micro-Computed Tomography (CT)

The maxillae of rats were obtained to detect bone parameters by micro-CT. Fixed in 4% paraformaldehyde, the bone morphometry was assessed using Skyscan1172 (Bruker, Kontich, Belgium) with an accuracy of 18 μm. Parameters including Bone volume/total volume (BV/TV), Bone surface/volume ratio (BS/BV) and Bone mineral density (BMD) were calculated.

### Isolation of Peripheral Blood Mononuclear Cells (PBMCs)

Peripheral blood from rats (5 ml per rat) was collected from abdominal aorta in the presence of heparin as the anticoagulant. Three milliliters of the whole blood was diluted with sterile PBS of the same volume and gently resuspended. Six milliliters of the diluted whole blood fraction was overlaid onto 3 ml of the Ficoll-Paque Plus (GE Healthcare Bio-Sciences Corp., Piscataway, NJ, United States) and then subjected to 800 × g for 20 min at RT with the centrifuge brake “off.” Then PBMC layers were washed twice with RPMI 1640 media by centrifugation at 1200 rpm for 5 min at 4°C. After isolation, all samples were dissolved in Trizol reagent (Takara, Kusatsu, Shiga, Japan) for lysis of cells to extract RNA.

### RNA Extraction and RT-PCR Analysis

The RNA was extracted from PBMCs and homogenization of cortex using Trizol reagent (Takara, Kusatsu, Shiga, Japan) and the Total RNA Kit (Omega Bio-Tek, Inc., Norcross, GA, United States), respectively. The purity and concentration of RNA, as well as the cDNA synthesis, were conducted according to Li et al. (2018). Subsequently, an RT-PCR assay was performed using SYBR Premix Ex Taq^TM^ (Takara, Kusatsu, Shiga, Japan) on a Roche LightCycler 480 Real-Time PCR Detection System (Roche, Basel, Switzerland) according to the manufacturer’s protocol. Data were then processed using the 2^–Δ^ ^Δ^ ^CT^ method. All results were based on at least three independent tests, and the final results were expressed as normalized fold values relative to the control group. The sequences of genes including Glyceraldehyde-3-phosphate dehydrogenase (GAPDH), IL-1β, IL-6, IL-8 and IL-21, APP, amyloid precursor-like protein 1 (APLP1), APLP2, a disintegrin and metalloproteinase 10 (ADAM10), β-site APP cleaving enzyme 1 (BACE1), presenilin 1 (PS1), PS2, TLR4, cluster of differentiation 14 (CD14) and their primer pairs were listed in Table 1.

**TABLE 1:** The sequences of genes and primer pairs.

**Target Gene**	**Primer Sequences**
GAPDH	Forward:5′-ACAGTCCATGCCATCACTGCC-3′
	Reverse:5′-GCCTGCTTCACCACCTTCTTG-3′
IL-1β	Forward:5′-AACCTGCTGGTGTGTGACGTTC-3′
	Reverse:5′-CAGCACGAGGCTTTTTTGTTGT-3′
IL-6	Forward:5′-GCCCTTCAGGAACAGCTATGA-3′
	Reverse:5′-TGTCAACAACATCAGTCCCAAGA-3′
IL-8	Forward:5′-CATTAATATTTAACGATGTGGATGCG-3′
	Reverse:5′-GCCTACCATCTTTAAACTGCACAAT-3′
IL-21	Forward:5′-GCTCCACAAGATGTAAAGGG-3′
	Reverse:5′-GTGCCTCTGTTTATTTCCTG-3′
APP	Forward:5′-AGAGGTCTACCCTGAACTGC-3′
	Reverse:5′-ATCGCTTACAAACTCACCAACT-3′
APLP1	Forward:5′-TCAGGTCTGCTGATCATGGGAGC-3′
	Reverse:5′-TGGGTGGGGAAGAGGACTTTATTG-3′
APLP2	Forward:5′-CAGAGCGACAGACCCTCATTC-3′
	Reverse:5′-TCTACTCGGGCCAAATGGGT-3′
ADAM10	Forward:5′-GCCTATGTCTTCACGGACCG-3′
	Reverse:5′-TGCCAGACCAAGAACACCATC-3′
BACE1	Forward:5′-CGGGAGTGGTATTATGAAGTG-3′
	Reverse:5′-AGGATGGTGATGCGGAAG-3′
PS1	Forward:5′-GAGGAAGACGAAGAGCTGACAT-3′
	Reverse:5′-GAAGCTGACTGACTTGATGGTG-3′
PS2	Forward:5′-GAGCAGAGCCAAATCAAAGG-3′
	Reverse:5′-GGGAGAAAGAACAGCTCGTG-3′
TLR4	Forward:5′-AGCCATTGCTGCCAACATCA-3′
	Reverse:5′-GCCAGAGCGGCTACTCAGAAAC-3′
CD14	Forward:5′-CTCAACCTAGAGCCGTTTCT-3′
	Reverse:5′-CAGGA TTGTCAGACAGGTCT-3′

### Measurements of Interleukins, Aβ and LPS by ELISA

Of the approximately 5 ml of blood collected as previously described, 2 ml of blood were collected in heparinized tubes for the measurement of plasma cytokines. After centrifuging (4°C, 2500 rpm*15 min), supernatant medium was separated and immediately aliquoted into 1.5 ml cryogenic tubes and frozen at –80°C until use. For tissue, radioimmunoprecipitation assay (RIPA) lysis buffer (Beyotime, Beijing, China), 1% protease inhibitor cocktail (Sigma, St. Louis, MO, United States), and 1% PMSF (Beyotime, Beijing, China) were used to homogenize samples of the cerebral cortex. Protein qualification was performed by BCA Protein Assay Kit (Beyotime, Beijing, China). Equal amounts of protein were used in ELISA to measure levels of IL-1β, IL-6, IL-8, IL-21 (UBI, Sunnyvale, CA, United States) and Aβ1-40, Aβ1-42 (Enzyme-linked Biotechnology, Shanghai, China) both in the cortex and plasma according to the manufacturer’s instructions. Level of LPS in the plasma of rats was detected by ELISA as well (SAB, College Park, Maryland, United States).

### Western Blot

The samples of the cerebral cortex of rats in four groups were homogenized and lysed by RIPA containing 1% protease inhibitor cocktail and 1% PMSF (Beyotime, Shanghai, China). Equal amounts of protein were separated by SDS polyacrylamide gel electrophoresis and transferred onto PVDF membrane blocked with 5% skimmed milk as previously described (Li et al., 2018). A pre-stained protein marker (Thermo Fisher Scientific, MA, United States) was run in parallel to detect the molecular weight of proteins. Proteins were probed with appropriate antibodies including anti-TLR4 (1:1000, abs132000; Absin Bioscience Inc., Shanghai, China), anti-CD14 (1:500, abs121538; Absin Bioscience Inc., Shanghai, China), anti-IRAK1 (1:1000, abs143411; Absin Bioscience Inc., Shanghai, China), anti-p65 (1:1000, no. 8242; Cell Signaling Technology, United States), anti-pp65 (1:1000, no.3033; Cell Signaling Technology, United States) anti-BACE1 (1:1000, no. 5606; Cell Signaling Technology, United States), anti-APP (1:1000, no. 2452; Cell Signaling Technology, United States) and anti-GAPDH (1:1000, AB-P-R001, Goodhere Biotechnology Co., Hangzhou, China). The data were quantified using the Image Studio Lite ver. 5.2 software.

### Immunohistochemistry

Rats were anesthetized with 10% chloral hydrate and perfused with cool PBS before removal of the brain. One hemisphere was placed in 4% paraformaldehyde overnight at 4°C, after which paraffin sections were prepared. This procedure is consistent with the previous study (Zhang et al., 2018). Briefly, brain sections were incubated with 3% H_2_O_2_ in methanol, blocked with 10% goat serum and incubated overnight at 4°C with the following primary antibodies: Ionized calciumbinding adaptor molecule 1 (Iba1) (1:400, ARG63338; Arigo Biolaboratories, Hsinchu City, Taiwan, China), Glial fibrillary acidic protein (GFAP) (1:400, ab7260; Abcam) to label microglia and astrocytes, anti-beta Amyloid1-42 antibody (1:50, ab10148; Abcam), anti-Tau antibody (1:400, ab32057; Abcam) and anti-APP antibody (1:100, MAB348; Millipore). After being washed, sections were incubated with biotinylated goat anti-rabbit or goat secondary antibody (1:200; Vector Laboratories, Burlingame, CA, United States). Images were obtained with a Leica camera.

The quantification of the immunohistochemical analysis for Iba1, GFAP, Aβ1-42, APP and Tau positive cells was performed by Image J software. The endpoints and process length were evaluated, and the numbers of positive cells were determined according to previous studies (Zhang et al., 2017; Young and Morrison, 2018). The average of the individual measurements was used to calculate group means.

### Statistical Analysis

All data are presented as the mean ± standard error of the mean (SEM). *P*-values were calculated with one-way ANOVA and two-way ANOVA with the GraphPad Prism software. An analysis of variance was performed using Turkey’s *post-hoc* multiple comparison test. A value of *p* < 0.05 was indicative of statistical significance.

## Results

### Assessment of Alveolar Bone Resorption

**FIGURE 1:**
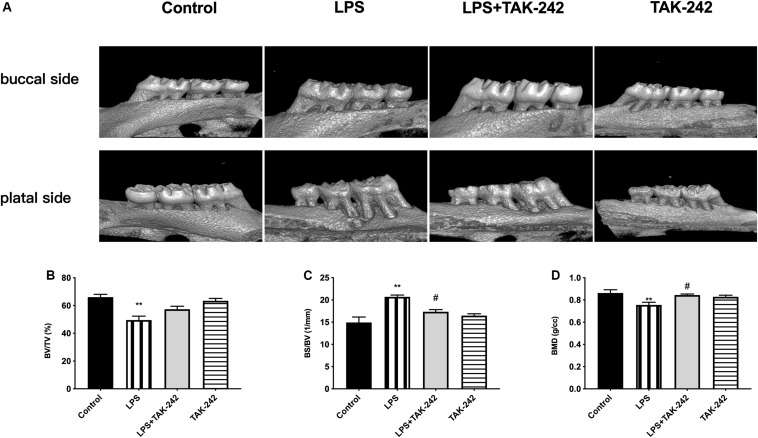
Effects of topical application of *P. gingivalis*-LPS into the gingival sulcus of maxillary first molars on alveolar bone. **(A)** Images captured by micro-CT scanning of molar region. Bone density analysis of alveolar bone including **(B)** BV/TV, one-way ANOVA, *F* = 13.65, **p < 0.01 compared to the control group. **(C)** BS/BV, one-way ANOVA, *F* = 13.35, **p < 0.01 compared to the control group and ^#^*p* < 0.05 compared to the LPS group. **(D)** BMD, one-way ANOVA, *F* = 6.979, **p < 0.01 compared to the control group and ^#^*p* < 0.05 compared to the LPS group (*n* = 4–6 per group).

As shown in Figure 1A, *P. gingivalis*-LPS led to posterior maxillary bone loss. There was a decrease in BV/TV and BMD (66.13 ± 1.98% vs. 49.56 ± 2.83% and 0.8743 ± 0.02 g/cc vs. 0.755 ± 0.02 g/cc, respectively) and an increase in BS/BV (14.92 ± 1.22/mm vs. 20.7 ± 0.40/mm) in the LPS group, which was reversed by TAK-242 (Figures 1B–D). This indicated that *P. gingivalis*-LPS could induce bone resorption similar to that in periodontitis. Moreover, these changes induced by *P. gingivalis*-LPS could be alleviated by TAK-242.

### Effects of Periodontal Inflammation on Locomotor Activity

**FIGURE 2:**
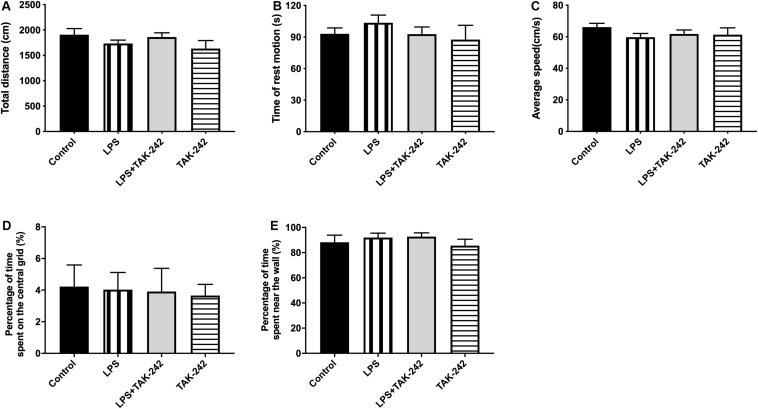
Effects of periodontal inflammation on animal activities. The open field test (OFT) was used to evaluate the locomotor activities of rats 3 days after the final administration **(A–E)**: **(A)** Total distance; one-way ANOVA, *p* = 0.3170. **(B)** Time of rest motion; one-way ANOVA, *F* = 0.2203, *p* = 0.8812. **(C)** Average speed. *F* = 0.8259, *p* = 0.4950. **(D)** Percentage of time spent on the central (%); one-way ANOVA, *F* = 0.03944, *p* = 0.9891. **(E)** Percentage of time spent near the wall; one-way ANOVA, *F* = 0.5656, *p* = 0.6455. Overall, no significant differences were observed between groups in OFT (*n* = 4–6 per group).

We used the OFT to assess whether periodontitis induced by *P. gingivalis*-LPS with/without intraperitoneal administration of TAK-242 could affect spontaneous activity in rats. As shown in Figure 2, there was no significant among-group difference in the behaviors, including total distance covered (Figure 2A), time of rest motion (Figure 2B), average speed (Figure 2C), percentage of time spent on the central (%) (Figure 2D), percentage of time spent near the wall (%) (Figure 2E). This indicated that the locomotor activity of the rats was not affected by *P. gingivalis*-LPS or TAK-242.

### Effects of Periodontal Inflammation on Spatial Learning and Memory

**FIGURE 3:**
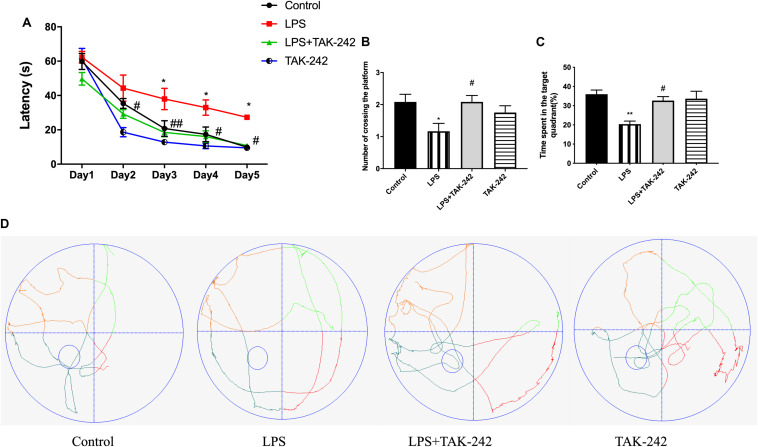
Effects of periodontal inflammation on spatial learning and memory. After the OFT, the MWM test was conducted to assess the learning and memory ability. **(A)** Latency to find the platform during the acquisition phase of the MWM test; two-way ANOVA, *F* = 1.154, *p < 0.05 compared to the control group and ^#^*p* < 0.05, ^##^*p* < 0.01 compared to the LPS group. **(B)** Number of platform crossings in the target quadrant; one-way ANOVA, *F* = 3.662, *p < 0.05 compared to the control group and ^#^*p* < 0.05 compared to the LPS group. **(C)** Percentage of time spent in the target quadrant; one-way ANOVA, *F* = 7.356, *p < 0.05 and **p < 0.01 compared to the control group, ^#^*p* < 0.05 compared to the LPS group. The LPS group was compared to the control group while the LPS+TAK-242 group was compared to the LPS group. **(D)** The typical trajectories of the LPS group approximated the arc of a circle, without any crossings over the original platform (platform is represented by a small circle) (*n* = 4–6 per group).

We used the MWM test to determine whether topical application of *P. gingivalis*-LPS into the palatal gingival sulcus of the maxillary first molars could affect learning and memory ability in rats. There was a chronological latency decrease in all the groups over the 5-day training period (Figure 3A). In the LPS group, the escape latency was significantly longer than it was in the control group at days 3, 4, and 5. There were no significant differences between the control group and the LPS+TAK-242 group. We removed the platform on the sixth day and found a significant decrease in the number of platform crossings in the target quadrant and the percentage of time spent in the target quadrant in the LPS group (Figures 3B,C). During the probe test, rats in the control group learned to directly navigate to the quadrant containing the hidden platform; however, this was not observed in the LPS group (Figure 3D). The changes mentioned above could be restored by TAK-242 administration. This indicated that periodontitis induced by *P. gingivalis*-LPS could be an important risk factor for learning and memory impairment.

### Effects of Periodontal Inflammation on the Plasma Levels of LPS and Inflammatory Cytokines

**FIGURE 4:**
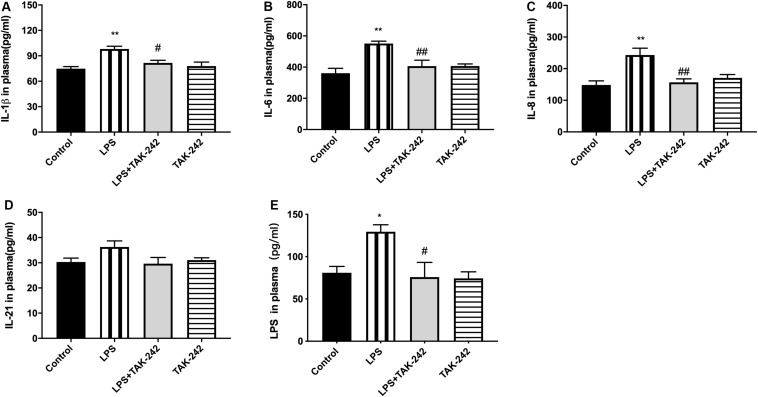
Effects of periodontal inflammation on level of LPS and inflammatory cytokines in plasma. ELISA was performed to detect level of LPS and protein expression of inflammatory cytokines in plasma. The protein level of cytokines in plasma in each group **(A–D)** according to a one-way ANOVA analysis: IL-1β (*F* = 8.168, *p* = 0.0012), IL-6 (*F* = 8.005, *p* = 0.0020), IL-8 (*F* = 8.358, *p* = 0.0010), IL-21 (*F* = 2.25, *p* = 0.1275). The level of LPS in plasma **(E)** according to a one-way ANOVA analysis: *F* = 5.750, *p* = 0.0112 (*n* = 4–6 per group, one-way ANOVA, *p < 0.05 and **p < 0.01 compared to the Control group, ^#^*p* < 0.05 and ^##^*p* < 0.01 compared to the LPS group).

Plasma levels of IL-1β, IL-6, IL-8, IL-21, and LPS were significantly higher in the LPS group than in the control group. Compared to the LPS group, the LPS+TAK-242 group showed reduced levels of IL-1β, IL-6, IL-8, and IL-21 proteins and LPS (Figure 4).

### Effects of Periodontal Inflammation on Microglia and Astrocytes in the Cortex

**FIGURE 5:**
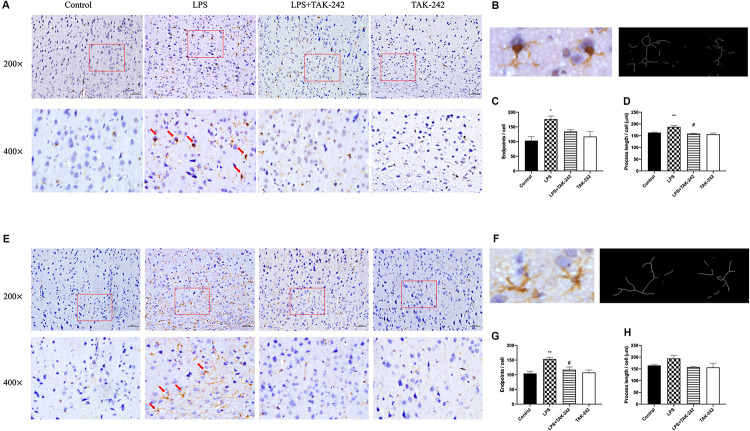
Effects of periodontal inflammation on microglia and astrocytes in the cortex. Histopathological analysis of brain sections was performed using immunohistochemistry. As shown in **(A,B)**, activated microglia labeled by Iba1 with irregular protrusions were observed in cortex of the LPS group, while a similar situation could be found in astrocytes labeled by GFAP **(E,F)**. The changes mentioned above could be attenuated by TAK-242 (200× and 400×, bar = 200 μm). The endpoints **(C,G)** and process length **(D,H)** according to a one-way ANOVA analysis: endpoints (*F* = 6.219, *p* = 0.0174) and process length (*F* = 10.74, *p* = 0.0025) of microglia, endpoints (*F* = 8.354, *p* = 0.0076) and process length (*F* = 2.595, *p* = 0.1249) of astrocytes (*n* = 3–4 per group, one-way ANOVA, *p < 0.05 and **p < 0.01 compared to the Control group, ^#^*p* < 0.05 compared to the LPS group).

As shown in Figure 5, activation of microglia and astrocytes could be found in the LPS group. Activated microglia, which are characterized by irregular protrusions and increased volume of cell bodies, were positively stained with Iba1. Activated microglia were observed in the cortex of the LPS group but rarely in the control group while activated astrocytes were positively stained with GFAP (Figures 5A,E). A relative increase in endpoints and process length was found, presenting as volume hypertrophy and irregular protrusions (Figures 5B–D,F–H). Changes mentioned above could be reversed by TAK-242 administration. These findings indicate that periodontitis induced by *P. gingivalis*-LPS could play an important role in neuroinflammation.

### Effects of Periodontal Inflammation on Inflammatory Cytokine Levels in the Cortex

**FIGURE 6:**
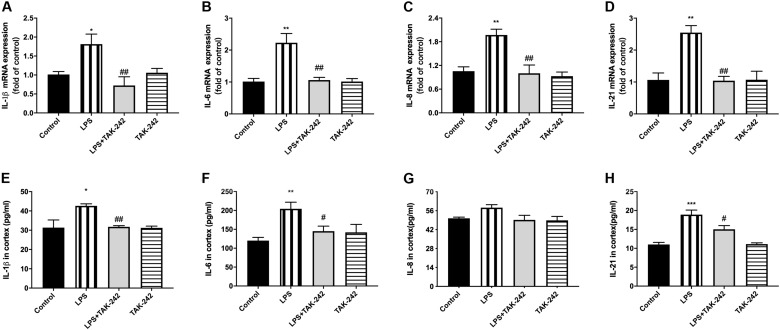
Effects of periodontal inflammation on protein expression of inflammatory cytokines in cortex. RT-PCR was performed to detect mRNA levels of IL-1β (*F* = 6.861, *p* = 0.0045), IL-6 (*F* = 11.05, *p* = 0.0007), IL-8 (*F* = 9.223, *p* = 0.0013), IL-21 (*F* = 11.89, *p* = 0.0007) in cortex of rats, while these changes were relieved by TAK-242 **(A–D)**. ELISA was performed to detect protein levels in cortex **(E–H)** in each group according to a one-way ANOVA analysis: IL-1β (*F* = 7.045, *p* = 0.0020), IL-6 (*F* = 7.145, *p* = 0.0019), IL-8 (*F* = 3.866, *p* = 0.0248), IL-21 (*F* = 15.22, *p* < 0.0001) (*n* = 4–6 per group, one-way ANOVA, *p < 0.05, **p < 0.01, and ***p < 0.001 compared to the Control group, ^#^*p* < 0.05 and ^##^*p* < 0.01 compared to the LPS group).

Compared to the control group, there were significantly higher mRNA and protein levels of IL-1β, IL-6, IL-8, and IL-21 in the cortex in the LPS group. Compared to the LPS group, the LPS+TAK-242 group showed reduced levels of IL-1β, IL-6, and IL-8 proteins (Figure 6). These findings demonstrate that periodontitis induced by *P. gingivalis*-LPS increases the expression of inflammatory factors in the central nervous system, which is significantly prevented by TAK-242.

### Effects of Periodontal Inflammation on APP Processing and Tau

**FIGURE 7:**
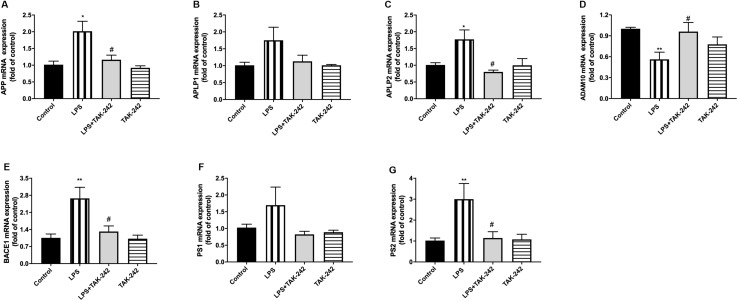
Effects of periodontal inflammation on mRNA level of APP processing. RT-PCR was performed to detect mRNA levels of APP, APLP1, and APLP2 on genes in comparison to the control group, while these changes were reversed by TAK-242 **(A–C)**. The mRNA expression of APP and the homologs in cortex in each group according to a one-way ANOVA analysis: APP (*F* = 6.327, *p* = 0.0049), APLP1 (*F* = 3.13, *p* = 0.0549), APLP2 (*F* = 6.611, *p* = 0.0052). mRNA levels of APP secretases including α-, β-, and γ-secretase were also upregulated, while these changes were reversed by TAK-242 **(D–G)**. The mRNA expression of secretases in cortex in each group according to a one-way ANOVA analysis: ADAM10 (*F* = 5.377, *p* = 0.0094), BACE1 (*F* = 7.911, *p* = 0.0021), PS1 (*F* = 2.292, *p* = 0.1227), PS2 (*F* = 5.18, *p* = 0.0142) (*n* = 4–6 per group, one-way ANOVA, *p < 0.05 and **p < 0.01 compared to the Control group and ^#^*p* < 0.05 compared to the LPS group).

**FIGURE 8:**
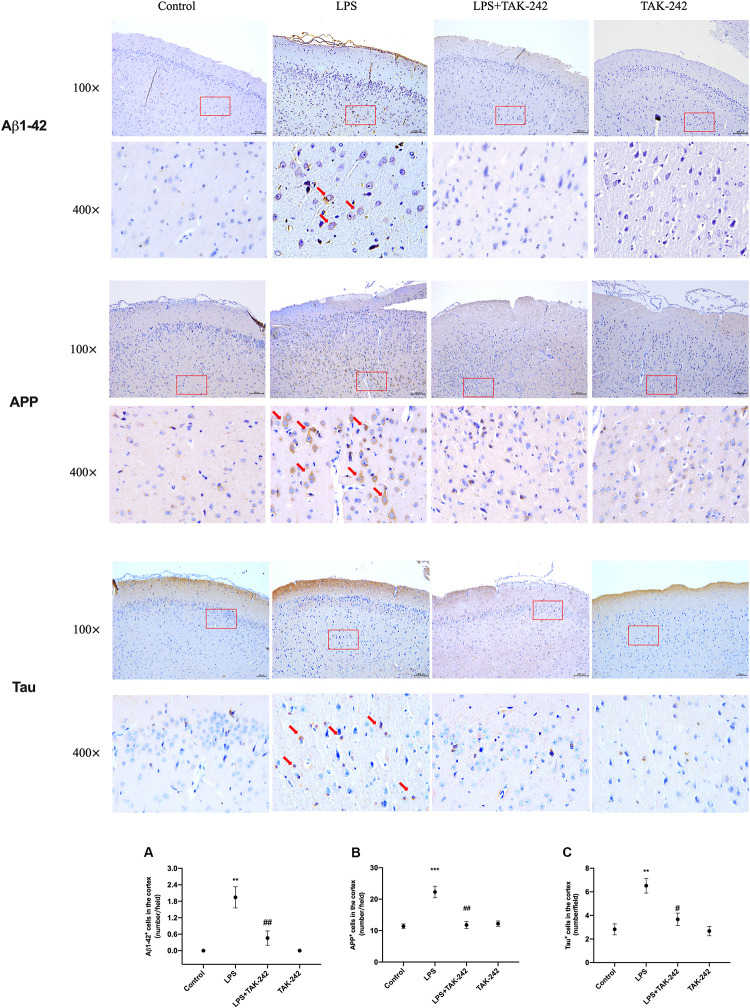
Effects of periodontal inflammation on expression of Aβ1-42, APP and Tau in the cortex. Histopathological analysis of brain sections was performed using immunohistochemistry. As shown in Figure 8, the positive expression of Aβ1-42 and APP in cytoplasm was observed in the LPS group, presenting as cytoplasmic yellow/brown particles. The number of Aβ1-42, APP, and Tau positive cells was increased. The changes mentioned above could be attenuated by TAK-242. Quantification of Aβ1-42, APP and Tau levels in cortex are shown, including **(A)** Aβ1-42 positive cells (number/field) (*F* = 15.59, *p* = 0.0011); **(B)** APP positive cells (number/field) (*F* = 18.9, *p* = 0.0005), and **(C)** Tau positive cells (number/field) (*F* = 12.35, *p* = 0.0023) (*n* = 3 per group, one-way ANOVA, **p < 0.01 and ***p < 0.001 compared to the Control group, ^#^*p* < 0.05 and ^##^*p* < 0.01 compared to the LPS group) (100× and 400×, bar = 400 μm).

**FIGURE 9:**
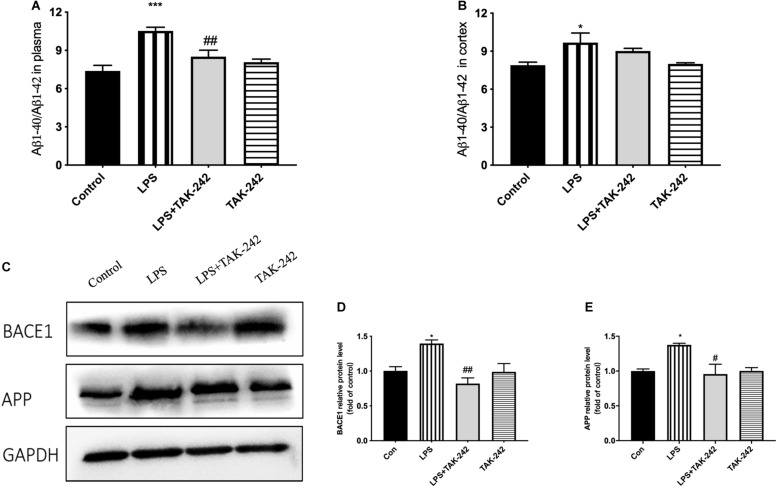
Effects of periodontal inflammation on the Aβ ratio and protein level of APP processing. ELISA was performed to detect the Aβ ratio (Aβ1-40/Aβ1-42) in plasma and cortex **(A,B)**. Periodontitis induced by *P. gingivalis*-LPS increased expression of the Aβ ratio in comparison to the control group, while these changes were reversed by TAK-242. According to a one-way ANOVA analysis, the Aβ ratio in plasma (*F* = 11.39, *p* = 0.0003), the Aβ ratio in cortex (*F* = 4.186, *p* = 0.0304). Protein level of BACE1 and APP in cortex was measured by western blot analysis **(C)**. Both proteins were upregulated by *P. gingivalis*-LPS and could be inhibited by TAK-242. The quantification of protein expression in each group according to a one-way ANOVA analysis **(D,E)**: BACE1 (*F* = 8.201, *p* = 0.0080), APP (*F* = 5.751, *p* = 0.0214). *p < 0.05, **p < 0.01 and ***p < 0.001 compared to the Control group and ^#^*p* < 0.05 and ^##^*p* < 0.01 compared to the LPS group.

Periodontitis induced by *P. gingivalis*-LPS increased the expression of APP and its homologs (APLP1 and APLP2) (Figures 7A–C). Further, it induced an increase in BACE1 and PS2 mRNA expression. In the control group, there was a decrease in ADAM10 mRNA expression (Figures 7D–G). Increased intracellular levels of Aβ1-42 and APP were observed in the LPS group, presenting as cytoplasmic yellow/brown particles (Figure 8). Besides, detection of Tau showed similar results. The number of positive cells were increased in the LPS group (Figures 8A–C). All the changes mentioned above could be alleviated by TLR4 inhibitor. The Aβ ratio (Aβ1-40/Aβ1-42) was upregulated in both the plasma and cortex (Figures 9A,B). Moreover, we measured BACE1 and APP protein expression in the cortex to confirm the increase in β-site APP cleaving in the LPS group (Figures 9C–E). Compared with the LPS group, the LPS+TAK-242 group showed reduced mRNA and protein expression related to APP processing.

### Role of the TLR4/NF-κB Signaling Pathway

**FIGURE 10:**
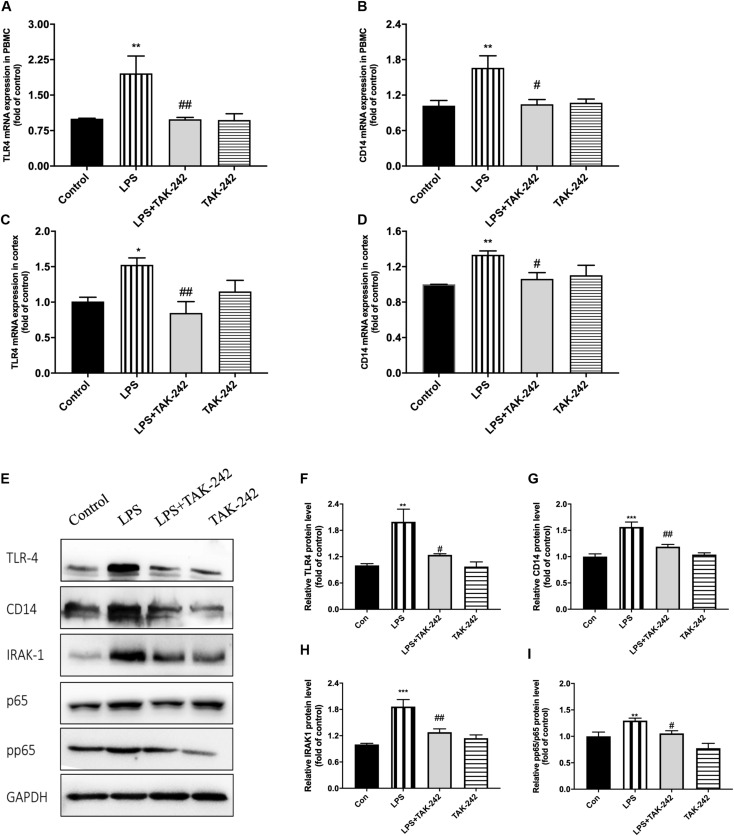
Periodontal inflammation could induce neuroinflammation via the activation of TLR4/NF-κB signaling pathway. RT-PCR was performed to detect mRNA levels of TLR4 and CD14 in PBMCs **(A,B)** and cortex **(C,D)**. RNA expression of TLR4 and CD14 was both upregulated by *P. gingivalis*-LPS; however, similar effects were not observed in LPS plus TAK-242 group. The mRNA expression in PBMCs in each group according to a one-way ANOVA analysis: TLR4 (*F* = 9.042, *p* = 0.0008), CD14 (*F* = 6.004, *p* = 0.0061) **(A,B)**. The mRNA expression in cortex in each group according to a one-way ANOVA analysis: TLR4 (*F* = 5.346, *p* = 0.0105), CD14 (*F* = 5.186, *p* = 0.0117) **(C,D)**. Expression of TLR4, CD14, IRAK1, p65, and pp65 was further measured by western blot analysis **(E)**. All above proteins was upregulated by *P. gingivalis*-LPS. High expression of these proteins was effectively inhibited by TAK-242. **(F–I)** The quantification of related protein expression in each group according to a one-way ANOVA analysis: TLR4 (*F* = 10.85, *p* = 0.0024), CD14 (*F* = 18.54, *p* = 0.0003), IRAK1 (*F* = 15.4, *p* < 0.0011), pp65 (*F* = 17.59, *p* = 0.0003) (*n* = 4–6 per group, one-way ANOVA, *p < 0.05, **p < 0.01, and ***p < 0.001 compared to the Control group, ^#^*p* < 0.05 and ^##^*p* < 0.01 compared to the LPS group).

Results of RT-PCR revealed that periodontal inflammation significantly increased the mRNA levels of TLR4 and CD14. Compared to the control group, the LPS group showed 2-fold and 1.5-fold increases in TLR4 and CD14 mRNA expression in the PBMCs and the cortex, respectively, which could be reversed by TAK-242 (Figures 10A–D). As shown in Figures 10E–J, we conducted western blot analysis to assess the underlying mechanisms of the neuroinflammation induced by periodontitis. We observed increased expression of TLR4, CD14, IRAK1, p65, and pp65 in the cortex of rats in the LPS group, which was reduced by TAK-242 administration. This indicated that periodontitis induced neuroinflammation through TLR4/NF-κB pathway cascades.

## Discussion

In this study, we demonstrated the association between periodontitis induced by topical application of *P. gingivalis*-LPS and learning and memory impairment *in vivo*. Micro-CT showed alveolar bone resorption. The MWM test showed that periodontitis impaired learning and memory ability. There was an increase in cortical and peripheral blood levels of inflammatory factors, as well as TLR4/NF-κB signaling pathway activation, which could be reversed by the TLR4 inhibitor TAK-242. Significant microglia and astrocyte activation were found in the cortex, which induced neuroinflammation. Furthermore, APP processing could be involved in this progress.

The association between systemic infection/inflammation and cognitive impairment has been reported (Zhang et al., 2018; Nie et al., 2019). In the present research, topical application of *P. gingivalis*-LPS into the gingival sulcus of maxillary first molars was used to cause periodontal inflammation according to a previous study (Yoshinaga et al., 2012). The most significant difference was the way of drug administration. Previous studies mostly used intraperitoneal injection that induced systemic inflammation, while we established periodontitis via topical application into the gingival sulcus. As the result of Micro-CT showed, a decrease in the trabecular bone volume fraction and bone mineral density could be found. This model was more similar to clinical periodontitis, considering it could mimic the damage of junctional epithelium and the formation of periodontal pocket (Bosshardt, 2018).

The locomotor activity of rats were not affected by topical application of *P. gingivalis*-LPST according to the results of the OFT. During the MWM test, however, the learning and memory ability could be impaired by periodontitis. Behavioral assessments demonstrated that topical application of *P. gingivalis*-LPS could induce learning and memory impairment. To investigate the association more comprehensively, more cognitive evaluation should be included in behavioral assessments using AD models in the following studies.

Periodontitis elicits a significant inflammatory response (Wu and Nakanishi, 2014). It has been proposed that peripheral inflammation/infection may be not only a contributor but also indeed a key determinant of the cognitive decline (Sochocka et al., 2017). Recent studies have shown that systemic infection/inflammation could induce cognitive impairment through neuroinflammation (Wu et al., 2017; Zhang et al., 2018). In periodontitis, local pro-inflammatory cytokines can enter the circulation, in turn contributing to atherosclerosis or exacerbating intra-uterine inflammation (Paraskevas et al., 2008). Patients with severe periodontitis have increased systemic inflammation (elevated cytokines such as IL-6) compared with healthy controls, whereas treatment of periodontitis reduces inflammation load (Friedewald et al., 2009). Similarly, we found significantly increased inflammatory cytokine expression (IL-1β, IL-6, IL-8, and IL-21) in the peripheral blood, which could be alleviated by TAK-242.

Neuroinflammation could play a significant role in cognitive dysfunction (Lyman et al., 2014). Inflammation in the brain is mainly caused by microglia and astrocyte activation, as well as the release of cytokines, chemokines or growth factors, which typically occur prior to cognitive dysfunction (Michelucci et al., 2009). We observed stimulation of microglia and astrocytes, which were labeled with Iba1 and GFAP, respectively, from a resting state to an activated state, which might have had toxic effects. Activated microglia could secret proinflammatory mediators (Schwab and McGeer, 2008; Venneti et al., 2008). Over-activated astrocytes could induce the production of inflammatory cytokines, which subsequently causes neuronal death and contributes to cognitive decline (Choi et al., 2016; Lian et al., 2016). Among these cytokines, IL-1β is the key molecule involved in neuroinflammation, which stimulates the release of multiple inflammatory mediators by activated microglia and leads to self-propagating neuroinflammation (Lai et al., 2017; Mendiola and Cardona, 2018). Regarding IL-6, an upstream IL-21 target, has been reported to enhance neuronal damage (Ringheim et al., 1998; Qiu and Gruol, 2003). IL-8 upregulation could be a very early event in neurodegeneration (Arosio et al., 2004; Galimberti et al., 2006). We found a positive correlation between levels of these cytokines in periphery and cortex. Therefore, we speculated that periodontitis could induce learning and memory impairment through neuroinflammation, which presented as microglial and astrocyte activation. Similar results could be found in previous studies (Martin et al., 2001; Liu et al., 2008; Terrando et al., 2010).

As one of the main triggers of inflammatory response, not only could the TLR4 signaling pathway regulate peripheral inflammation, but also neuroinflammation (Laflamme and Rivest, 2001). Specifically, TLR4 plays an important role in microglial neurotoxicity given that it is activated by LPS binding to initiate signal transduction and pro-inflammatory cytokines (Walter et al., 2007). Moreover, increased expression of TLR4, CD14, IRAK1, and pp65/p65 could be attenuated by the TLR4 selective blocker TAK-242. Consistent with the findings of Zakaria et al. (2017), this suggests that *P. gingivalis*-LPS could stimulate brain secretion of inflammatory cytokines via TLR4 activation, which subsequently induces neuroinflammation.

Besides, activated TLR4 signaling pathway and dysregulated cytokines might stimulate inflammatory processes by affecting APP processing (Reed-Geaghan et al., 2009). Multiple proinflammatory cytokines have been shown to upregulate APP expression and BACE1 activity in the brain (Brugg et al., 1995; Chen et al., 2012). The APP family has two mammalian homologs; namely, APLP1 and APLP2 (Müller and Zheng, 2012; Müller et al., 2017). Several common features of the APP family members include processing by α-(ADAM10), β- (BACE1), and γ-secretase (PS1 and PS2) (Eggert et al., 2004; Li and Südhof, 2004). As a rate-limiting enzyme that initiates Aβ formation, BACE1 plays an essential role (Trojanowski et al., 1993). Expression of APP, BACE1, and PS1/2 was increased. All the changes mentioned above could be alleviated by TLR4 inhibitor. Our study demonstrated that periodontitis induced by *P. gingivalis* -LPS could facilitate abnormal APP processing. This indicated that periodontitis induced by *P. gingivalis*-LPS could modulate APP processing through enhanced β- and γ-site secretase activity, consistent with the increase of APP in patients with chronic periodontitis (Abe et al., 2011). According to our results, cytoplasmic yellow/brown particles could be found and intracellular levels of Aβ1-42 as well as APP were increased in the cortex of LPS group, indicating that periodontitis could induce abnormal APP processing. Due to the process of Aβ extracellular deposition may occur at a later time point due to severe neuronal dysfunction and degeneration, it was hard to find obvious Aβ1-42 plaques in the present model induced by LPS, similar to the results of Sheng et al. (2003) and Pintado et al. (2012).

Abnormal APP processing could generate Aβ, which plays a pivotal role in the pathogenesis of cognitive disorders. It has traditionally been thought that Aβ in the brain originates from the brain tissue itself (Bu et al., 2018). However, some have suggested that the source of cerebral amyloid may originate in the periphery, including platelets, liver monocytes/macrophages, skin fibroblasts, skeletal muscles and cerebrovascular smooth muscle cells (Citron et al., 1994; Li et al., 1995; Kuo et al., 2000; Van Nostrand and Melchor, 2001; DeMattos et al., 2002; Nie et al., 2019). Aβ produced in peripheral tissues or cells could be secreted into blood circulation and subsequently entered brain (Li et al., 1995; Bu et al., 2018; Nie et al., 2019). Our results showed an increase in the ratio of Aβ1-40/Aβ1-42 in both plasma and cortex, which was in concordance with Anantharaman et al. (2006). We speculated that periodontitis could directly/indirectly expand Aβ pools in periphery and thereby contributes to the increase of Aβ in the brain.

In this work, we established a periodontitis model and indicated the association between periodontitis and learning and memory impairment. Increase of inflammatory factors, activation of microglia and astrocytes and abnormal APP processing were found, suggesting that periodontitis might be a possible contributor to AD-like pathology. However, AD models should be used in following researches for further assessment of AD-pathologies and more sophisticated evaluation of association between periodontitis and AD.

## Conclusion

Our findings indicated that periodontitis induced by topical application of *P. gingivalis*-LPS could contribute to learning and memory impairment via neuroinflammation induced by TLR4/NF-κB signaling pathway activation in SD rats. Furthermore, abnormal APP processing could be involved in this progress. Therefore, periodontitis could not only affect the teeth-supporting structures but also cause a significant inflammatory load in both the peripheral blood and central nervous system, which leads to learning and memory impairment.

## Data Availability Statement

The raw data supporting the conclusions of this article will be made available by the authors, without undue reservation, to any qualified researcher.

## Ethics Statement

The animal study was reviewed and approved by the Animal Care and Welfare Committee of Shanghai Jiao Tong University School of Medicine.

## Author Contributions

YH, WZ, and ZS wrote the main manuscript text. YH, HL, JZ, XZ, XX, and CQ performed the research. YH, HL, YL, and HC analyzed the data. WZ and ZS designed the main research, provided the necessary guidance on the performance of all the experiment, and contributed the essential reagents and tools. All authors read and approved the final manuscript.

## Conflict of Interest

The authors declare that the research was conducted in the absence of any commercial or financial relationships that could be construed as a potential conflict of interest.

## Supplementary Material

FIGURE S1 Congo red staining showed that LPS group rats’ cortex had sporadic amyloid deposition.
