# Supplementary figures and images for: Periodontitis Induced by P. gingivalis-LPS Is Associated With Neuroinflammation and Learning and Memory Impairment in Sprague-Dawley Rats
Source: Front Neurosci. 2020 Jul 2;14:658. doi: 10.3389/fnins.2020.00658 (PMC7344110; doi:10.3389/fnins.2020.00658)

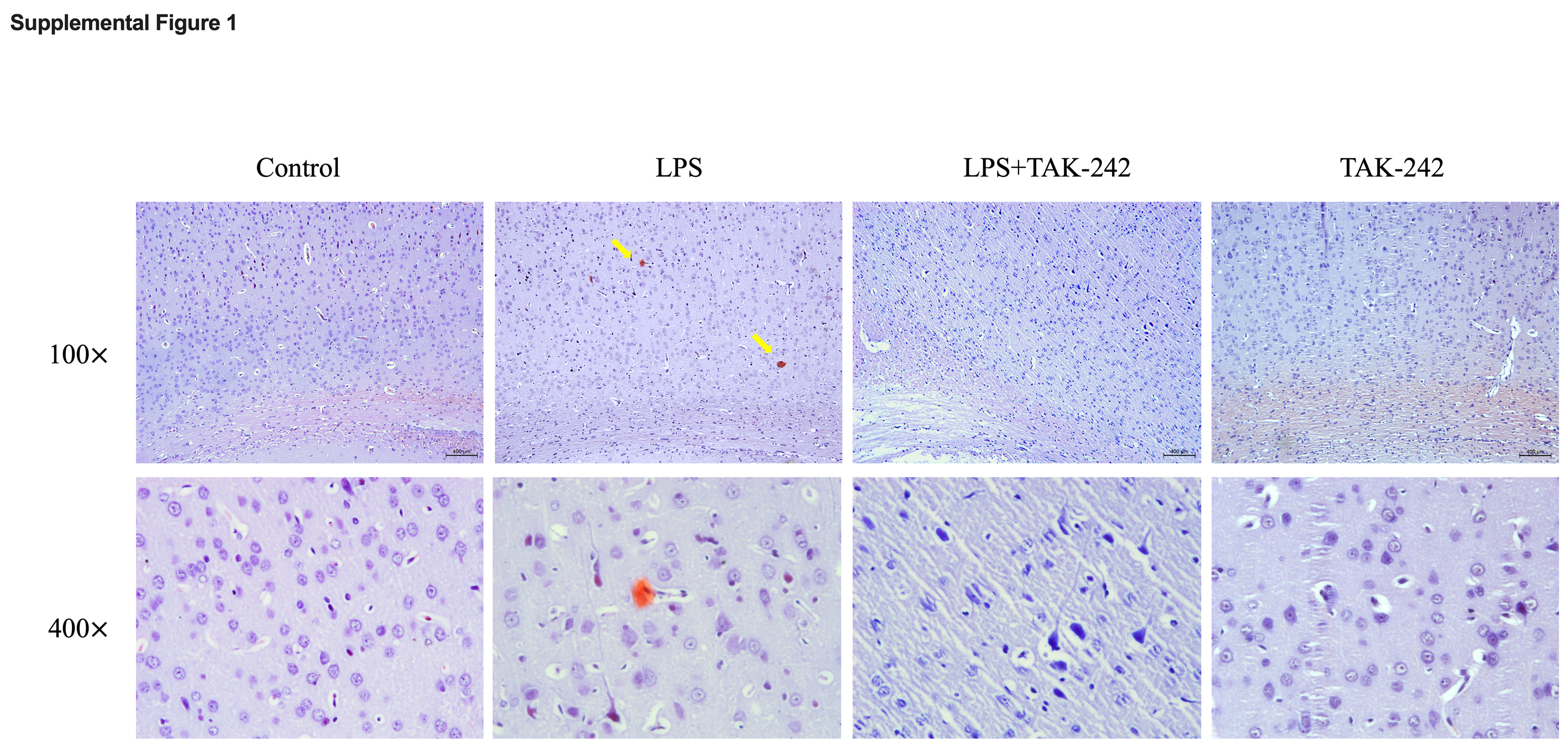

Supplement: FIGURE S1 — Congo red staining showed that LPS group rats’ cortex had sporadic amyloid deposition. [file Image_1.TIFF]
